# An Effective Baseline Correction Algorithm Using Broad Gaussian Vectors for Chemical Agent Detection with Known Raman Signature Spectra

**DOI:** 10.3390/s21248260

**Published:** 2021-12-10

**Authors:** Hyeong Geun Yu, Dong Jo Park, Dong Eui Chang, Hyunwoo Nam

**Affiliations:** 1 School of Electrical Engineering, Korea Advanced Institute of Science and Technology, Daejeon 34141, Korea; elloss@kaist.ac.kr (H.G.Y.); djpark@kaist.ac.kr (D.J.P.); 2 Chem-Bio Technology Center, Agency for Defense Development, Daejeon 34188, Korea; hyunwoonam@add.re.kr

**Keywords:** Raman spectroscopy, signal processing, chemical agent detection, baseline correction, generalized likelihood ratio test

## Abstract

Raman spectroscopy, which analyzes a Raman scattering spectrum of a target, has emerged as a key technology for non-contact chemical agent (CA) detection. Many CA detection algorithms based on Raman spectroscopy have been studied. However, the baseline, which is caused by fluorescence generated when measuring the Raman scattering spectrum, degrades the performance of CA detection algorithms. Therefore, we propose a baseline correction algorithm that removes the baseline, while minimizing the distortion of the Raman scattering spectrum. Assuming that the baseline is a linear combination of broad Gaussian vectors, we model the measured spectrum as a linear combination of broad Gaussian vectors, bases of background materials and the reference spectra of target CAs. Then, we estimate the baseline and Raman scattering spectrum together using the least squares method. Design parameters of the broad Gaussian vectors are discussed. The proposed algorithm requires reference spectra of target CAs and the background basis matrix. Such prior information can be provided when applying the CA detection algorithm. Via the experiment with real CA spectra measured by the Raman spectrometer, we show that the proposed baseline correction algorithm is more effective for removing the baseline and improving the detection performance, than conventional baseline correction algorithms.

## 1. Introduction

Many chemical agents (CAs) have been developed during the advancement of human civilization. Since many CAs, which are harmful when in contact with the human body, are colorless and odorless, it is difficult to respond to threats of chemical gas terror and chemical gas leak accident quickly. To deal with these threats, non-contact CA detection techniques are essential. As one of the non-contact CA detection techniques, the Raman spectrometer, which is capable of non-destructive analysis of target materials, has been studied [1,2,3]. When a light is irradiated onto a material, some fraction of light is scattered with some frequency change, which is called Raman scattering. Raman scattering depends on the molecular structure and characteristics of the material [4]. Then, a Raman spectrometer measures this Raman scattering and generates a spectrum. In a non-contact measurement setting, the measured spectrum contains not only the Raman (scattering) spectrum of the CA, but also those of background materials and noise. Accordingly, many CA detection algorithms using Raman spectroscopy have been studied [5,6,7,8,9,10].

However, CA detection algorithms are disturbed by the baseline in the measured spectrum. The baseline is mainly caused by fluorescence that occurs almost simultaneously with the generation of the Raman scattering [11]. In order to suppress the baseline, several methods of physically blocking the fluorescence signature have been proposed [12,13,14,15]. These methods are based on the fact that the lifetime of fluorescence is much longer than that of the Raman scattering. By shutting down the gate before fluorescence occurs, the fluorescence signature can be suppressed in the measured spectrum. However, it is hard to control the gate open time very precisely (about a $10^{-12}$ s scale). Therefore, the baseline correction algorithms, which estimate the baseline from the measured spectrum and remove it, have been proposed [16,17,18,19,20].

The baseline is usually a smooth curve in contrast to a Raman spectrum composed of sharp peaks. Filter-based algorithms were proposed [16,17]. Because these filter-based algorithms distort the Raman spectrum, penalized least squares(PLS)-based algorithms have been proposed [18,19,20]. These PLS-based algorithms estimate the baseline by smoothing a curve of the measured spectrum while giving a penalty to the signatures at Raman shifts suspected of having the Raman scattering. PLS-based algorithms distinguish the baseline from the Raman spectrum according to the difference in curvature. However, when the signal-to-noise ratio (SNR) of the measured spectrum is low, the curvature of the Raman spectrum is degraded and PLS-based algorithms calculate the incorrect baseline.

In this paper, to estimate the baseline while minimizing the distortion of the Raman spectrum, we propose the algorithm that estimates both the baseline and Raman spectrum simultaneously. Assuming that the baseline is a weighted summation of broad Gaussian vectors, we model the measured spectrum as a linear combination of broad Gaussian vectors, the reference CA spectrum, and bases of background materials. Then, we obtain coefficients of broad Gaussian vectors by the least squares method and calculate the baseline using these coefficients. From the experiment with real CA data measured by a Raman spectrometer (Korea Raman Agent Monitoring System, K-RAMS), we demonstrate that the proposed baseline correction algorithm accurately estimates the baseline while preserving the Raman spectrum of the CA and background. We also show that the proposed algorithm improves the CA detection performance better than other baseline correction algorithms.

There are three contributions in this article. The first contribution is to accurately estimate both the Raman signature and baseline using reference spectra of target CAs, background basis matrix and broad Gaussian vectors. The second contribution is to propose how to design the broad Gaussian vectors. We introduce some conditions for Gaussian vectors to estimate the baselines effectively. Based on these conditions, the mean and variance of each Gaussian vector are determined. The final contribution is to show the novelty of the proposed algorithm via experiments with real CA measurements.

The remainder of this paper is organized as follows. In Section 2, we introduce the system model for measured spectra and review the conventional baseline correction algorithms. In Section 3, we explain the proposed baseline correction algorithm that estimates the baseline and Raman signature simultaneously using broad Gaussian vectors, the reference spectrum of CA, and the basis function of background materials. We also discuss design parameters for broad Gaussian vectors. In Section 4, real-data experiments presenting the superiority of the proposed baseline correction algorithm are described. The final discussions are drawn in Section 5.

## 2. Conventional Baseline Correction Algorithms

Before we review the conventional baseline correction algorithms, we briefly introduce the signal model of the spectrum measured by the Raman spectrometer. Let $x={\left[x_{1},x_{2},\ldots ,x_{p}\right]}^{T}\in R^{p}$ denote the measured spectrum, where $x_{i}$ is a spectral value at the *i*th Raman shift, for i=1,…,p, and *p* is the number of channels. Here, $x\in R^{p}$ means that the vector x is a vector with *p* real values. Then, the measured spectrum x can be represented as a sum of the Raman spectrum, the baseline, and the noise [18,19,20,21] as

(1) $$\begin{array}{c} x=t+b+n, \end{array}$$

where $t\in R^{p}$ is the Raman spectrum, $b\in R^{p}$ denotes the baseline, and $n\in R^{p}$ is the noise signature. The noise emerged by the spectrometer is modeled as the Gaussian noise with mean $0\in R^{p}$ and covariance $\gamma \Sigma \in R^{p\times p}$, i.e., n∼N(0,γΣ). Here, Σ is a diagonal matrix of which diagonal components are $\left[\sigma_{1}^{2},\ldots ,\sigma_{p}^{2}\right]$, γ is the correction factor and $\Sigma \in R^{p\times p}$ implies that the matrix Σ has *p* real rows and *p* real columns.

In (1), the Raman spectrum t is represented as a linear combination of the reference spectra of target CAs and the background basis matrix [5] as follows:

(2) $$\begin{array}{c} t=\mathrm{Sg}+K_{bg}y_{bg}=\mathrm{Ky}, \end{array}$$

where $S\in R^{p\times C}$ is the reference CA matrix that consists of *C* reference CA spectra $s_{c}\in R^{p}$, for c=1,…,C, $K_{bg}=\left[k_{bg,1},\ldots ,k_{bg,M}\right]\in R^{p\times M}$ refers to the background basis matrix composed of *M* bases of the background materials $k_{bg,m}\in R^{p}$, for m=1,…,M. Then, $g\in R^{C}$ is the intensity vector for each CA signature, $y_{bg}={\left[y_{bg,1}^{T},\ldots ,y_{bg,M}^{T}\right]}^{T}\in R{}^{M}$ denotes the coefficient vector of the background basis matrix, $K=\left[S,K_{bg}\right]\in R^{p\times C+M}$, and $y={\left[g^{T},y_{bg}^{T}\right]}^{T}\in R^{C+M}$. Background basis functions $k_{bg,m}$ are obtained by measuring many Raman spectra for background materials, correcting Raman spectra, applying the singular value decomposition (SVD) to the corrected Raman spectra, and extracting singular vectors corresponding to large singular values [22].

Substituting (2) into (1), the measured Raman spectrum is expressed as

(3) $$\begin{array}{c} x=\mathrm{Ky}+b+n. \end{array}$$

Let $x^{\prime }\in R^{p}$ denote the baseline corrected spectrum, i.e., $x^{\prime }=x-b$. Then, the baseline corrected spectrum $x^{\prime }$ follows the linear subspace model (LSM) under two hypotheses as

(4) $$\begin{array}{cc} H_{0}:x^{\prime } & =K_{bg}y_{bg}+n,\\ H_{1}:x^{\prime } & =\mathrm{Ky}+n, \end{array}$$

where $H_{0}$ and $H_{1}$ represent hypotheses for the absence and presence of the CA, respectively. Because the Ky is a deterministic vector and n is a Gaussian random vector, the $x^{\prime }$ is also the Gaussian random vector. As we can see in (4), if the baseline remains in the spectrum $x^{\prime }$, the spectrum $x^{\prime }$ does not follow the LSM. It results in reducing the accuracy of the detection algorithms. To address this problem, many baseline correction algorithms have been proposed. We introduce these baseline correction algorithms.

### 2.1. Iterative Median Filter (IMF)

In general, the baseline is in the form of a smooth curve unlike the Raman signature composed of several peaks of certain shapes. The median filter constructs a window with *n* spectral values nearby a spectral value $x_{i}$ in the spectrum, finds the median value ${\hat{x}}_{i}$ in the window, and replace $x_{i}$ with ${\hat{x}}_{i}$. The median filter repeats this process for all spectral values and obtains the smoothing curve of the spectrum. The iterative median filter finds the baseline by applying the median filter iteratively.

### 2.2. Rolling Circle Filter (RCF)

The rolling circle filter (RCF) is an algorithm that estimates the baseline by rolling a circle of an appropriate size in contact with the measured spectrum. In the process of rolling the circle, the curvature radius of the circle is smaller than that of the baseline, but larger than that of the Raman spectrum so that the circle is tangent to the baseline, but not to the Raman spectrum. Finally, the baseline can be calculated by connecting arcs of the circles tangent to the baseline.

### 2.3. Asymmetric Least Squares (ALS)

Since these filter-based algorithms consider low frequency component signatures of the Raman spectrum as the baseline, they cause the distortion of the Raman spectrum during the baseline correction. To deal with this problem, algorithms based on the penalized least squares (PLS) have been proposed. These algorithms estimate the baseline using the least squares fitting while giving a penalty to the Raman shifts suspected of having Raman signatures. Let us define L(b) as the cost function according to the baseline b as follows:

(5) $$\begin{array}{c} L\left(b\right)={\left(x-b\right)}^{T}W\left(x-b\right)+\lambda b^{T}D^{T}\mathrm{Db}, \end{array}$$

where $W\in R^{p\times p}$ is a penalty matrix that is a diagonal matrix composed of a penalty $w_{i}$ at the *i*th Raman shift, for i=1,…,p, λ is a regularization coefficient for the smoothing, and $D\in R^{p\times p+2}$ is the secondary order difference matrix.

In (5), the term ${\left(x-b\right)}^{T}W\left(x-b\right)$ implies fitness of the baseline b to the spectrum x and $\lambda b^{T}D^{T}\mathrm{Db}$ represents smoothness of the baseline. The optimal baseline b is obtained by solving ∂L(b)/∂b=0 as

(6) $$\begin{array}{c} b={\left(W+\lambda D^{T}D\right)}^{-1}\mathrm{Wx}. \end{array}$$

In (6), the baseline b is determined by the measured spectrum x and the penalty matrix W. Let $i_{b}$ and $i_{R}$ denote the Raman shifts with and without the Raman spectrum, respectively. Then, the penalty $w_{i_{b}}$ is close to 1, otherwise, the penalty $w_{i_{R}}$ becomes almost 0. If we know Raman shifts at which the Raman signature exists, we obtain the penalty matrix W exactly. However, it is an unrealistic assumption to know these Raman shifts beforehand.

To estimate the baseline without the information about Raman shifts having the Raman spectrum, Eilers and Boelens proposed an asymmetric least squares (ALS) [18]. In the ALS, the penalty $w_{i}$ is allocated according to the baseline $b_{i}$ and measured spectral value $x_{i}$ at the *i*th Raman shift as follows:

(7) $$\begin{array}{c} w_{i}=\left\{\begin{array}{c} \alpha \;\;\;\;\;\;\;\;\;\;\;\;\;\;\;x_{i}\geq b_{i},\\ 1-\alpha \;\;\;\;\;\;\;\;\;x_{i}<b_{i}, \end{array}\right. \end{array}$$

where α represents the asymmetric parameter determining the penalty and is recommended to be assigned from $10^{-3}$ to $10^{-1}$. In the ALS, if the measured spectral value $x_{i}$ exceeds the baseline $b_{i}$, it is determined that there is the Raman spectrum at the *i*th Raman shift. From (6) and (7), the baseline b is not expressed in a closed-form solution. Therefore, we obtain the penalty matrix W and the baseline b using an iterative method until the penalty matrix does not change.

### 2.4. Adaptive Iterative Reweighted Penalized Least Squares (AirPLS)

In the ALS, penalties at Raman shifts with the Raman spectrum are all the same. Since the curvature of the Raman spectrum at each Raman shift varies, the penalty need to be changed according to the Raman shift. From this point of view, Zhang proposed the adaptive iterative reweighted penalized least squares (AirPLS) [19]. In the AirPLS, the penalty is determined by the difference between the measured spectrum and the baseline. At the *j*th iteration step, the penalty $w_{i}$ is obtained as

(8) $$\begin{array}{c} w_{i}=\left\{\begin{array}{c} 0\;\;\;\;\;\;\;\;\;\;\;\;\;\;\;\;\;\;\;\;\;\;\;\;\;\;\;\;\;\;\;\;\;\;x_{i}\geq b_{i},\\ \exp\left[j\left(x_{i}-b_{i}\right)\left|d^{-}\right|\right]\;\;\;\;\;\;x_{i}<b_{i}, \end{array}\right. \end{array}$$

where the vector $d^{-}$ consists of negative elements of d=x−b. Like the ALS, the baseline b and the penalty matrix W are obtained by alternating (6) and (8) iteratively. Here, the condition to terminate the iteration is as follows:

(9) $$\begin{array}{c} d<0.001\times \left|x\right|. \end{array}$$

### 2.5. Asymmetrically Reweighted Penalized Least Squares (ArPLS)

The ALS and AirPLS extract the baseline from the measured spectrum well while preserving the Raman signature. However, these algorithms are vulnerable to random noises. To deal with this problem, Baek proposed an asymmetrically reweighted penalized least squares (ArPLS) based on the partially balanced weighting scheme. The ArPLS acquires the mean and variance of noise signatures at Raman shifts without the Raman spectrum. Using these statistics, penalties at Raman shifts with the Raman spectrum are corrected as

(10) $$\begin{array}{c} w_{i}=\left\{\begin{array}{cc} \frac{1}{1+\exp\left[2\left(x_{i}-b_{i}+m_{d^{-}}-2\sigma_{d^{-}}\right)/\sigma_{d^{-}}\right]}\;\;\;\;\;\; & x_{i}\geq b_{i},\\ 1\;\;\;\;\;\;\;\;\;\;\;\;\;\;\;\;\;\;\;\;\;\;\;\;\;\;\;\;\;\;\;\;\;\;\;\;\;\;\;\;\;\;\;\;\;\;\;\;\;\; & x_{i}<b_{i}, \end{array}\right. \end{array}$$

where $m_{d^{-}}$ and $\sigma_{d^{-}}$ are the mean and the standard deviation of $d^{-}$. The baseline is acquired by alternating (6) and (10) iteratively.

## 3. Proposed Baseline Correction Algorithm

Conventional baseline correction algorithms estimate the baseline under the assumption that the curvature of the Raman signature is significantly larger than that of the baseline. However, when the measured Raman spectrum has a low signal-to-noise ratio (SNR), the curvature of the Raman signature is reduced, resulting in distortion of the Raman signature during the baseline correction. In this section, we propose a baseline correction algorithm that is more suitable to detection algorithms that exploits the background basis matrix $K_{bg}$ and the reference spectrum s of the target CA.

Since the baseline is generally a curve with less curvature, it is modeled as a linear combination of broad Gaussian vectors [23] as

(11) $$\begin{array}{c} b=K_{bl}y_{bl}, \end{array}$$

where $K_{bl}=\left[k_{bl,1},\ldots ,k_{bl,L}\right]\in R^{p\times L}$ is a broad Gaussian matrix composed of *L* broad Gaussian vectors $k_{bl,l}\in R^{p}$, for l=1,…,L, and $y_{bl}\in R^{L}$ is a coefficient vector for broad Gaussian vectors. Since widths of broad Gaussian vectors are wider than those of peaks in the Raman spectrum, $K_{bl}$ and K are linearly independent. By substituting (11) into (3), the measured spectrum x is represented as the LSM form as

(12) $$\begin{array}{c} x=\mathrm{Ky}+K_{bl}y_{bl}+n=K^{\prime }y^{\prime }+n \end{array}$$

where $K^{\prime }=\left[K,K_{bl}\right]$ and $y^{\prime }={\left[y^{T},y_{bl}^{T}\right]}^{T}$. To remove the baseline while minimizing the distortion of the Raman spectrum, we need to estimate both the baseline $K_{bl}y_{bl}$ and the Raman spectrum Ky simultaneously. It is accomplished by obtaining ${\hat{y}}^{\prime }$ via the least squares method as

(13) $$\begin{array}{c} {\hat{y}}^{\prime }={\left[{\left(K^{\prime }\right)}^{T}K^{\prime }\right]}^{-1}{\left(K^{\prime }\right)}^{T}x. \end{array}$$

Finally, using ${\hat{y}}_{bl}$ in ${\hat{y}}^{\prime }$, we remove the estimated baseline $\hat{b}=K_{bl}{\hat{y}}_{bl}$ from the measured spectrum x.

The accuracy of the estimated baseline depends on the broad Gaussian vector $k_{bl,l}\in R{}^{p}$, for l=1,…,L. The *i*th component $k_{bl,li}$ of the broad Gaussian vector $k_{bl,l}$ is expressed as the Gaussian function with mean $m_{l}$ and variance $\sigma_{l}^{2}$ as

(14) $$\begin{array}{c} k_{bl,li}=\exp\left[-\frac{1}{2\sigma_{l}^{2}}{\left(\nu_{i}-m_{l}\right)}^{2}\right], \end{array}$$

where $\nu_{i}$ is the wavenumber of the *i*th Raman shift. The mean $m_{l}$ determines the interval between the broad Gaussian vectors. In order to estimate a shape baseline generated at outer Raman shifts, it is recommended that the means of the first and last broad Gaussian vectors be set to wavenumbers of the first and last Raman shift, respectively, i.e., $m_{1}=\nu_{1}$ and $m_{L}=\nu_{L}$. If we do not follow this recommendation, coefficients of the first and last Gaussian vectors are so large that coefficient estimation for a shape baseline generated at outer Raman shifts becomes unstable. Therefore, we fix the means of the first and last broad Gaussian vectors. Let broad Gaussian vectors be equally spaced. Then, mean $m_{l}$ satisfying this condition is obtained as

(15) $$\begin{array}{c} m_{l}=\left(\nu_{L}-\nu_{i}\right)\cdot \frac{l-1}{L-1}. \end{array}$$

Then, the interval between broad Gaussian vectors is determined as the number of broad Gaussian vectors *L*.

**Figure 1:**
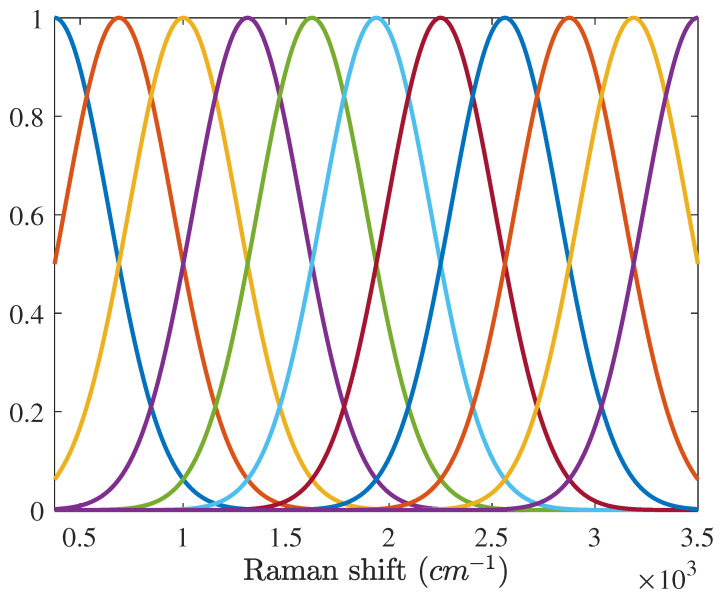
Eleven broad Gaussian vectors used in the experiment. The center interval of each Gaussian vector is 312 cm${}^{-1}$ and the variance of each Gaussian vector is 265 cm${}^{-1}$

The variance $\sigma_{l}^{2}$ implies the width of a broad Gaussian vector. It is recommended that the variance be set to 1/2ln2 times the interval between adjacent broad Gaussian vectors as

(16) $$\begin{array}{c} \sigma_{l}^{2}=\frac{1}{2\ln2}\Delta m=\frac{\nu_{L}-\nu_{1}}{2\ln2(L-1)} \end{array}$$

where $\Delta m[=m_{l+1}-m_{l}=\left(\nu_{L}-\nu_{1}\right)/\left(L-1\right)]$ denotes the interval between adjacent broad Gaussian vectors. As shown in (15) and (16), the mean $m_{l}$ and variance $\sigma_{l}^{2}$ are determined by the number of Gaussian vectors *L*. The larger the number of Gaussian vectors is used, the more accurate the baseline is estimated. However, when the number of Gaussian vectors exceeds a certain level and the width of the Gaussian vectors becomes narrower than that of peaks of the Raman signature, (12) is linearly dependent and both the Raman spectrum and baseline are over-fitted. This overfitting skews the estimation results for both the Raman signature and baseline.The width of each peak in the Raman spectrum is less than 350 cm${}^{-1}$ in general. Therefore, it is recommended that the variance of each broad Gaussian vector exceed 350/2ln2≈250 cm${}^{-1}$. In the experiment, we used 11 broad Gaussian vectors of which variances are 265 cm${}^{-1}$ when the wavenumber range of the Raman shift is from 375 to 3500 cm${}^{-1}$. These broad Gaussian function are shown in Figure 1.

In fact, the baseline can be modeled as a linear combinations of other basis functions, i.e., polynomial functions. Nevertheless, the broad Gaussian vectors have some benefits. The first benefit is easy to design broad Gaussian vectors. Design parameters for the broad Gaussian vectors are only two, i.e., mean and variance, and are determined by the number of broad Gaussian vectors *L*. The second benefit is that the broad Gaussian vectors do not cause Gibson errors. To estimate the baseline effectively, it is recommended that all basis functions have the same sign. When designing the basis function, all values below zero are set to 0, which makes the ringing artifacts that are mainly caused by discontinuity of basis functions [24]. On the other hand, the broad Gaussian vectors always have positive values and are free from ringing artifacts.

## 4. Experimental Results

In this section, we describe experiments using real CA data to compare baseline correction algorithms. Raman spectrum data used in the experiments were collected by the Korea Raman Agent Monitoring System (K-RAMS, Agency for Defense Development, Korea), which provides data with a resolution of 3.3 cm${}^{-1}$ from 375 to 3500 cm${}^{-1}$ with 947 channels. In the K-RAMS, a KrF excimer laser at 248.35 nm was used as the light source to generate Raman scattering of chemicals [25].

**Figure 2:**
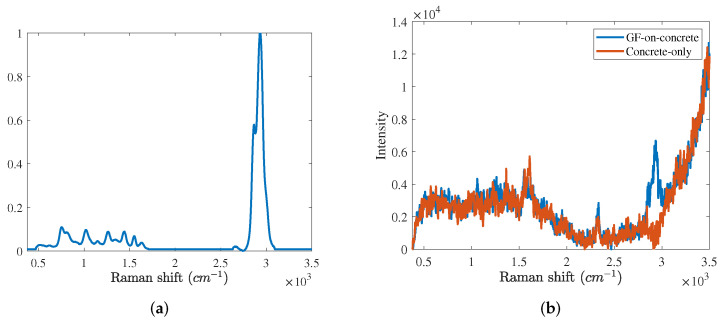
(**a**) Reference spectrum of the cyclosarin (GF), (**b**) GF-on-concrete and concrete-only spectra.

In the experiment, the cyclosarin (GF) was selected as a target chemical agent. Figure 2a shows the reference Raman spectrum of the GF. In Figure 2a, there are a main peak at 2700∼3100 cm${}^{-1}$ bands and several subpeaks at 500∼1700 cm${}^{-1}$ bands. The reference CA matrix consists of reference spectra of seven target CAs, i.e., the GF, distilled mustard (H), nitrogen mustard (HN), benzyl chloride, DMMP, MES, and phosphorus trichloride. The background basis matrix $K_{bg}$ is composed of six basis spectra of major background materials, i.e., the oxygen, nitrogen, concrete, asphalt, grass and soil. The molecular structures and reference Raman spectra of seven target CAs are introduced in [26].

The experiment conditions are as follows. The distance between the spectrometer and each target chemical was set to 1 m. We measured the concrete background 1000 times. Then, we dropped GF 0.5 μL on the concrete background and measured the GF 500 times. We denote concrete background and GF spectra as concrete-only spectra and GF-on-concrete spectra, respectively. Figure 2b shows the GF-on-concrete and concrete-only spectra. In the GF-on-concrete spectrum, the main peak of the GF is confirmed. However, subpeaks of the GF are obscured by noise signatures. Since Raman spectra were taken at a very close range (about under 10 cm) in general contact measurements, the signal-to-noise ratio (SNR) of the chemical agent (CA) was so high that every subpeak is well observed. However, for the non-contact measurements (about more than 0.5 m), some fractions of Raman scattering are measured by the Raman spectrometer. In both spectra, peaks of the oxygen and nitrogen are represented at 1550 and 2300 cm${}^{-1}$ bands, respectively. We also see the baseline throughout the entire band.

**Figure 3:**
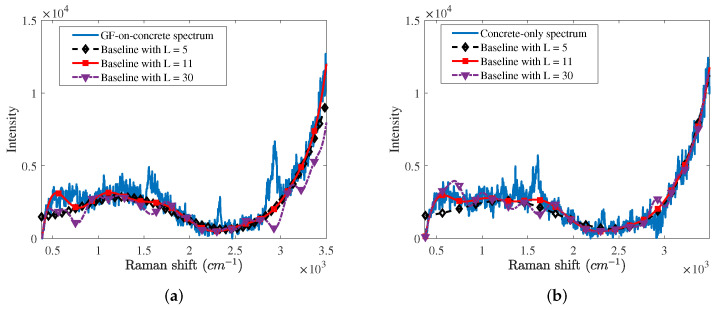
Baselines estimated by the proposed algorithm with 5, 11 and 30 broad Gaussian vectors from (**a**) the GF-on-concrete spectrum and (**b**) the concrete-only spectrum.

First, we compared baselines estimated by the proposed algorithm according to the number of Gaussian vectors as shown in Figure 3. We applied the proposed algorithm with 5, 11, and 30 Gaussian vectors, i.e., L=5,11 and 30, to the GF-on-concrete and concrete-only spectra. In cases of L=5 and 11, the proposed algorithm well estimates the baseline except for Raman spectrum signatures, such as the peaks of the oxygen, nitrogen, and GF. It is confirmed that the baseline with L=11 is more accurate than that with L=5. However, the proposed algorithm with L=30 does not approximate the baseline due to an overfitting and causes the distortion of the Raman spectrum.

For more objective competition, we adopt the root mean square modeling error (RMSME), which is a metric evaluating how baseline correction algorithms effectively removes the baseline while preserving the Raman spectrum. The modeling error n is determined from the baseline-corrected spectrum $x^{\prime }$ as

(17) $$\begin{array}{c} n=x^{\prime }-K\hat{y}, \end{array}$$

where the estimated coefficient vector $\hat{y}$ is obtained by the least squares method as $\hat{y}={\left[K^{T}K\right]}^{-1}K^{T}x^{\prime }$. Then, the RMSME is defined as follows:

(18) $$\begin{array}{c} RMSME=\sqrt{\frac{1}{p}\sum_{i=1}^{p}n_{i}^{2}}, \end{array}$$

where $n_{i}$ is the *i*th value of the modeling error n.

Table 1 describes RMSME averages of 500 GF-on-concrete spectra and 1000 concrete-only spectra according to the number of broad Gaussian vectors. In Table 1, ‘Non BC’ indicates the measured spectra without any baseline correction algorithms. The RMSMEs of spectra without the baseline corrections are higher than those with the proposed baseline correction algorithm. In case of L=11, modeling errors are minimized, which implies the proposed baseline correction algorithm with L=11 accurately estimates the baseline while preserving the Raman spectrum as much as possible.

Next, we compared the proposed baseline correction algorithm with other baseline correction algorithms mentioned in Section 2, i.e., the iterative median filter (IMF), rolling circle filter (RCF), asymmetric least squares (ALS), adaptive iterative reweighted penalized least squares (AirPLS), and asymmetrically reweighted penalized least squares (ArPLS). We found the optimal design parameters for each algorithm, which minimizes the RMSME numerically. The optimal design parameters for each algorithm are as follows. In the case of the IMF, the window size is 300cm${}^{-1}$ and the number of iterations is 5. For the RCF, the radius of the circle is set to 100cm${}^{-1}$. The regularization parameters of the ALS, AirPLS, and ArPLS are determined as 1000, 50, and 200, respectively. In the case of the proposed algorithm, the optimum number of Gaussian vectors is 11.

**Figure 4:**
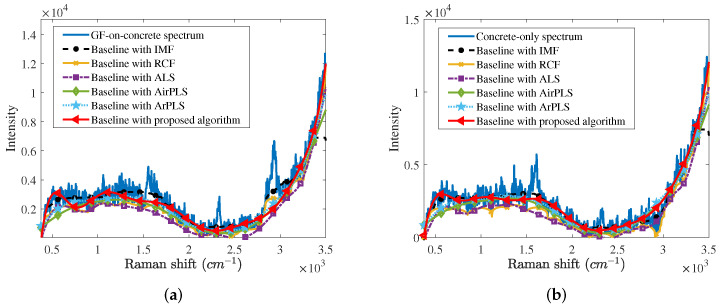
Baselines estimated by several baseline correction algorithms from (**a**) the GF-on-concrete spectrum and (**b**) the concrete-only spectrum.

Figure 4a,b depict baselines estimated by several baseline correction algorithms from the GF-on-concrete spectrum and concrete-only spectrum, respectively. In cases of IMF and RCF, a little Raman spectrum of the GF at 2700∼3100 cm${}^{-1}$ band is regarded as the baseline. The baseline estimated by the ALS is located below the other baselines since the ALS is affected by the negative part of the noise. On the other hand, AirPLS, ArPLS, and the proposed baseline correction algorithm estimate the baseline.

For more objective comparisons, we also obtained RMSME averages of 500 GF-on-concrete spectra and 1000 concrete-only spectra for baseline correction algorithms as shown in Table 2. It is confirmed that any baseline correction algorithms can suppress the modeling error. Since IMF and RCF distort peaks of GF shown in Figure 4, RMSMEs for IMF and RCF are less than those for other baseline correction algorithms. The proposed algorithm minimizes the RMSMEs, because the proposed algorithm preserves the Raman spectrum as much as possible by estimating the baseline and Raman spectrum simultaneously.

Finally, we analyze the effect of each baseline removal algorithm on the CA detection performance using the receiver of characteristic (ROC) curve. The ROC curve, which shows the relation between false alarm probabilities and detection probabilities, is widely used for a metric for evaluating the detection performance. In the experiment, we selected the adaptive subspace detector (ASD) as a CA detection algorithm. The ASD, which is known as the optimal detector for the LSM [22,27], is obtained by applying the generalized likelihood ratio test (GLRT) to (4).

The test statistic $T_{ASD}\left(x^{\prime }\right)$ of the ASD is defined as

(19) $$\begin{array}{c} T_{ASD}\left(x^{\prime }\right)=\frac{{\left(x^{\prime }\right)}^{T}P_{K_{bg}}^{⊥}x^{\prime }-{\left(x^{\prime }\right)}^{T}P_{K}^{⊥}x^{\prime }}{{\left(x^{\prime }\right)}^{T}P_{K}^{⊥}x^{\prime }}, \end{array}$$

where $T_{ASD}\left(x^{\prime }\right)$ denotes the test statistic of the ASD for the baseline-corrected spectrum $x^{\prime }$, $P_{K_{bg}}^{⊥}=I-K_{bg}{\left(K_{bg}^{T}K_{bg}\right)}^{-1}K_{bg}^{T}\in R{}^{p\times p}$ and $P_{K}^{⊥}=I-K{\left(K^{T}K\right)}^{-1}K^{T}\in R{}^{p\times p}$ are the orthogonal projection matrices for a subspace spanned by K and $K_{bg}$, respectively. Here, K and $K_{bg}$ denote the Raman signature basis matrix and background signature basis matrix, respectively, as described in Section 2. If $T_{ASD}\left(x^{\prime }\right)$ exceeds a detection threshold β, it is determined that the hypothesis $H_{0}$ is true. Otherwise, $H_{1}$ is true.

**Figure 5:**
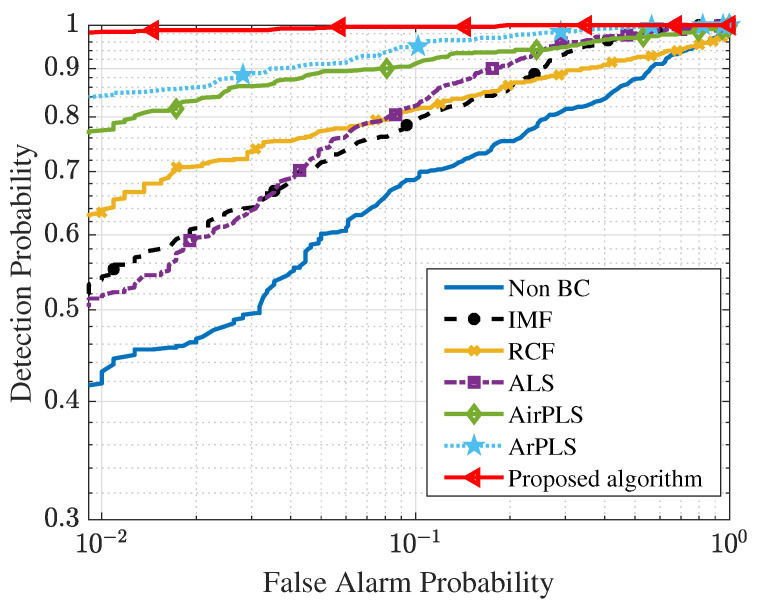
ROC curves of the ASD for the GF detection.

Then, we applied the ASD to baseline corrected spectra and obtained ROC curves. To acquire the ROC curves, 500 GF-on-concrete spectra and 1000 concrete-only spectra were used. Figure 5 presents the ROC curves of the ASD according to the baseline correction algorithms. The closer the ROC curve is to the upper left, the better detection performance is, since it has the higher detection probability under the same false alarm probability. It can be seen that the detection performance is good in order of the proposed algorithm, ArPLS, AirPLS, ALS, IMF, RCF, and non-baseline correction. This result is in agreement with the result pertaining to the RMSME averages in Table 2.

**Figure 6:**
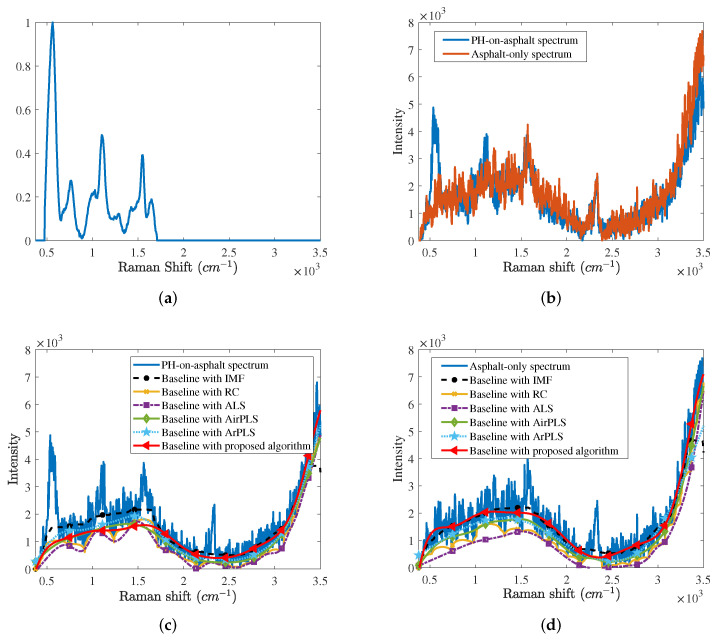
(**a**) Reference spectrum of the phosphorus trichloride (PH) and (**b**) PH-on-asphalt and asphalt-only spectra. Baselines estimated by several baseline correction algorithms from (**c**) the PH-on-asphalt spectrum and (**d**) the asphalt-only spectrum.

We conducted another experiment with a phosphorus trichloride (PH) on the asphalt background. First, we graphically compared the baseline correction results according to several baseline correction algorithms. Figure 6a shows the reference Raman spectrum of the PH. In Figure 6a, there are a main peak at the 450∼650 cm${}^{-1}$ band and several subpeaks at the 650∼1800 cm${}^{-1}$ band. The experiment conditions are almost the same as the GF experiment. We measured the asphalt background 1600 times. Then, we dropped 2 μL of the PH on the asphalt background and measured the PH 500 times. We denote asphalt background and PH spectra as asphalt-only spectra and PH-on-asphalt spectra, respectively.

Figure 6b shows the PH-on-asphalt and asphalt-only spectra. In the PH-on-asphalt spectrum, the main peak of the PH is confirmed, however, some subpeaks of the PH are obscured by noise signatures. We also see the baseline throughout the entire band. Figure 6c,d depict baselines estimated by several baseline correction algorithms from the PH-on-asphalt spectrum and asphalt-only spectrum, respectively. Like Figure 4a,b, the AirPLS, the ArPLS, and the proposed baseline correction algorithm estimate the baseline.

Next, we also obtain the RMSME averages of 500 PH-on-asphalt spectra and 1600 asphalt-only spectra for baseline correction algorithms as shown in Table 3. Except that RMSMEs of the IMF are higher than those of the RCF, the overall trend is the same as Table 2. RMSMEs of the proposed algorithm are lower than other algorithms, which indicates that the proposed algorithm most accurately removes the baseline while preserving the Raman signal.

**Figure 7:**
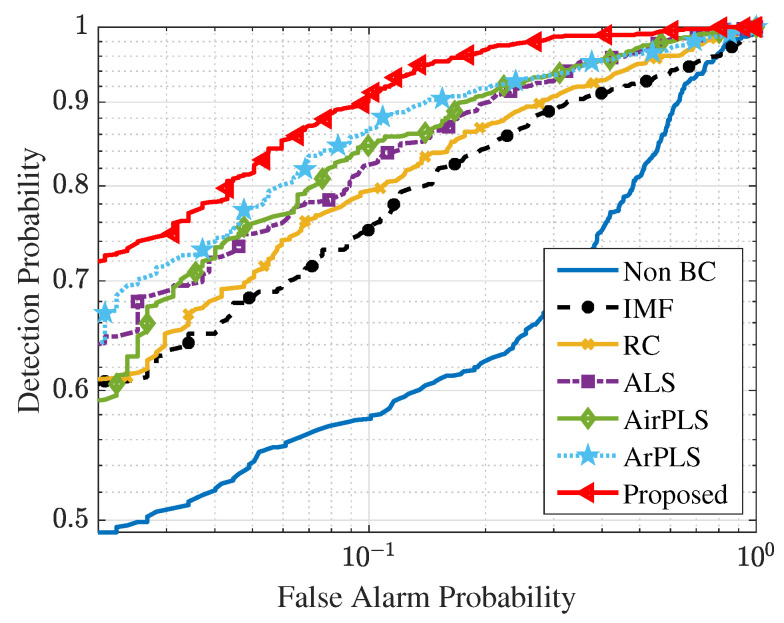
ROC curves of the ASD for the PH detection.

Finally, we acquired the ROC curves for each baseline correction algorithm with 500 PH-on-asphalt spectra and 1600 asphalt-only spectra, as shown Figure 7. It can be seen that the proposed algorithm greatly improves the detection performance of the ASD. This result is in agreement with the result pertaining to the RMSME averages in Table 3. Through these experiments, it is confirmed that the proposed baseline correction algorithm improves the detection performance of the ASD more than the other baseline correction algorithms.

## 5. Conclusions

Raman spectroscopy is a method for non-contact detection of chemical agents (CAs). The baseline, which is mainly caused by fluorescence, degrades the CA detection performance. Many baseline correction algorithms have been proposed; however, these algorithms cause the distortion of the Raman spectrum. To remove the baseline while minimizing the distortion of Raman signatures, we proposed an algorithm that estimates the baseline and Raman spectrum together using the background basis matrix and reference spectra of target CAs, which are essential for CA detection algorithms. Assuming that baseline is represented as a linear combination of broad Gaussian vectors, we obtained the coefficients of the baseline and Raman spectrum using the least squares method. Then, we estimated the baseline using the coefficients of the baseline and removed the baseline from the measured spectrum.

In the experiments using the CA spectra measured by the real Raman spectrometer, the proposed baseline correction algorithm effectively removed the baseline. It is confirmed that the proposed baseline correction algorithm improved the detection performance better than other baseline correction algorithms. The proposed baseline correction algorithm will be applied not only in the field of Raman spectroscopy but also in other fields that employ the linear subspace model, which assumes that reference spectra of target CAs and the background basis matrix are already known. The proposed algorithm has a limitation that the reference spectra of target CAs and the background basis matrix are required to estimate baseline. To overcome this limitation, a new algorithm built on the deep neural network will be needed in the near future.

## Figures and Tables

**Table 1:** RMSME averages of the proposed algorithm according to the number of Gaussian vectors.

	Non BC	L=5	L=11	L=30
GF-on-concrete	1203.4	492.51	471.83	558.83
Concrete-only	880.55	307.71	298.40	314.98

**Table 2:** RMSME averages of 500 GF-on-concrete spectra and 1000 concrete-only spectra for baseline correction algorithms.

	Non BC	IMF	RCF	ALS	AirPLS	ArPLS	Proposed
GF-on-concrete	1203.4	607.01	635.53	555.96	528.96	533.84	471.83
Concrete-only	880.55	400.10	419.63	350.16	328.47	323.43	298.40

**Table 3:** RMSME averages of 500 PH-on-asphalt spectra and 1600 asphalt-only spectra for baseline correction algorithms.

	Non BC	IMF	RCF	ALS	AirPLS	ArPLS	Proposed
PH-on-asphalt	887.78	518.12	429.10	408.18	390.67	342.60	327.71
Asphalt-only	658.59	425.71	361.89	318.15	300.81	297.47	259.79

## Data Availability

The data are not publicly available due to military security.
